# Pulmonary delivery of triptolide-loaded liposomes decorated with anti-carbonic anhydrase IX antibody for lung cancer therapy

**DOI:** 10.1038/s41598-017-00957-4

**Published:** 2017-04-20

**Authors:** Congcong Lin, Blenda Chi Kwan Wong, Hubiao Chen, Zhaoxiang Bian, Ge Zhang, Xue Zhang, Muhammad Kashif Riaz, Deependra Tyagi, Ge Lin, Yanbo Zhang, Jinjin Wang, Aiping Lu, Zhijun Yang

**Affiliations:** 1 School of Chinese Medicine, Hong Kong Baptist University, 7 Baptist University Road, Kowloon Tong, Hong Kong China; 2 Changshu Research Institute, Hong Kong Baptist University, Changshu Economic and Technological Development (CETD) Zone, Changshu, 215500 China; 3 School of Biomedical Sciences, Chinese University of Hong Kong, Area 39, CUHK, Shatin, NT, Hong Kong China; 4 School of Chinese Medicine, Li Ka Shing Faculty of Medicine, The University of Hong Kong, 10 Sassoon Road, Pokfulam, Hong Kong China

## Abstract

Antibody-decorated liposomes can facilitate the precise delivery of chemotherapeutic drugs to the lung by targeting a recognition factor present on the surface of lung tumor cells. Carbonic anhydrase IX (CA IX) is an enzyme expressed on the surface of lung cancer cells with a restricted expression in normal lungs. Here, we explored the utility of anti-carbonic anhydrase IX (CA IX) antibody, conjugated to the surface of triptolide (TPL)-loaded liposomes (CA IX-TPL-Lips), to promote the therapeutic effects for lung cancer via pulmonary administration. It was found that the CA IX-TPL-Lips significantly improved the cellular uptake efficiency in both CA IX-positive human non-small cell lung cancer cells (A549) and A549 tumor spheroids, resulting in the efficient cell killing compared with free TPL and non-targeted TPL-Lips. *In vivo*, CA IX-Lips via pulmonary delivery showed specificity and a sustained release property resided up to 96 h in the lung, both of which improved the efficiency of TPL formulations in restraining tumor growth and significantly prolonged the lifespan of mice with orthotopic lung tumors. The results suggest that CA IX-decorated liposomes can potentially be used as an effective therapeutic strategy for lung cancer.

## Introduction

Lung cancer is one of the most common lethal malignancies worldwide with 1.59 million deaths each year^1^. Although the treatment of lung cancer has vastly progressed during the last 50 years, the survival rate remains low^2^. Non-small cell lung cancer (NSCLC) is the most common lung cancer (85%) and has been linked to poor prognosis with 5-year survival rates of only 15%^3^. Low accumulation of therapeutic agents in the tumor site, and fear of high-dose treatment due to toxicity with severe adverse effects are the main obstacles in efficient lung cancer therapy. We hypothesize that the efficiency of lung cancer therapy will be substantially enhanced by delivering chemotherapeutic agents directly to the lung targeting specific receptors. Pulmonary delivery of anti-cancer drugs could provide a better retention of active ingredients in the lung and minimize adverse effects on healthy organs by limiting drug concentration in the blood^2^. Simultaneously targeting specific antigens or receptors confined to cancerous cells with drug delivery carriers will increase the accumulation of the drug in lung cancer cells and enhance its lethality for cancerous cells without disturbing normal lung tissue.

Receptors on cancer cells can recognize biological ligands or antibodies on the liposomal surface, allowing for the selective and precise delivery of drugs to the proposed site without disturbing normal cells^4–6^. Thus, identifying specific receptors or antigens restricted to cancer cells is essential. Carbonic anhydrase IX (CA IX) is a hypoxia-inducible enzyme controlled via the hypoxia-inducible factor (HIF)^7^. Hypoxia is a salient feature of many types of solid tumors, as a core cellular response to this microenviromental stress, CA IX is overexpressed in different types of cancers including cancers of the lung^8–10^, kidney^11^, colon^12^, and breast^13^. It has been reported that CA IX mRNA was expressed in 100% of NSCLC^14^. In contrast, it has a restricted expression pattern in normal tissue^15^. In addition, localization of the N-terminal active site of CA IX on the tumor cell surface allows efficient targeting by antibodies or small molecule inhibitors^16^. These unique features of CA IX have made it a promising target for lung cancer therapy.

CA IX has been known as a potential cancer treatment target, by using CA IX inhibitors and monoclonal antibody based drugs. Most of the CA IX inhibitors are sulfonamide-based compounds and so far, only SLC-0111 is accessed to Phase I clinical trial for the treatment of solid tumor^17, 18^. The ‘off-target’ effect, potent allergic reaction and myotoxicity problem of CA IX inhibitors has been an arduous challenge for successful clinical application^19^. It is worth noting that CA IX specific monoclonal antibodies (mAbs) with high binding affinity and specificity are the predominant agent in clinical development. As the most extensively studied mAb, cG250 demonstrates antibody-mediated cell cytotoxicity, positive impact on disease burden and good tolerance in Phase I and II trials^20^. But, it failed to show a significant improvement of disease free survival rate in Phase III trials for the treatment of clear cell renal cell carcinoma^21^. Moreover, no successful study for the treatment of lung cancer is reported using anti-CA IX antibody. Apart from using CA IX inhibitors and mAbs based drugs for anti-cancer treatment, we used anti-CA IX antibody as a targeting ligand onto liposome surface to deliver therapeutic payloads. This approach facilitates the targeted delivery to the tumor site and further enhances the therapeutic response of the drugs. So far, *in vivo* efficacy using anti-CA IX antibody modified liposomes for lung cancer therapy has not been performed, and this is also the premise of the study.

In addition, recent studies have shown that CA IX is being detected in the body fluid of lung cancer patients due to ectodomain shedding^22^. The targeting accumulation of immune-therapeutics in tumor site will be impacted by the noneffective binding in the circulation. To avoid this problem, we utilized pulmonary administration in this study. It provides the possibility of regional drug delivery to the lung, which leads to the high drug concentration to the tumor site comparatively to in the blood and further enhances the targeting efficacy.

Triptolide (TPL) is an active drug against NSCLC^23–25^. It is isolated from the Chinese herb *Tripterygium wilfordii Hook F* ^26^. Previous experiments have shown that TPL is not only cytotoxic to lung cancer cells but also sensitizes them to chemotherapy *in vitro* ^27, 28^. However, its therapeutic potential has been limited by the poor water solubility (0.017 mg/mL) and high toxicity (LD_50_, 0.8 mg/kg)^26, 29^. Thus, it is desirable to explore strategies for facilitating TPL targeting to human NSCLC.

**Figure 1:**
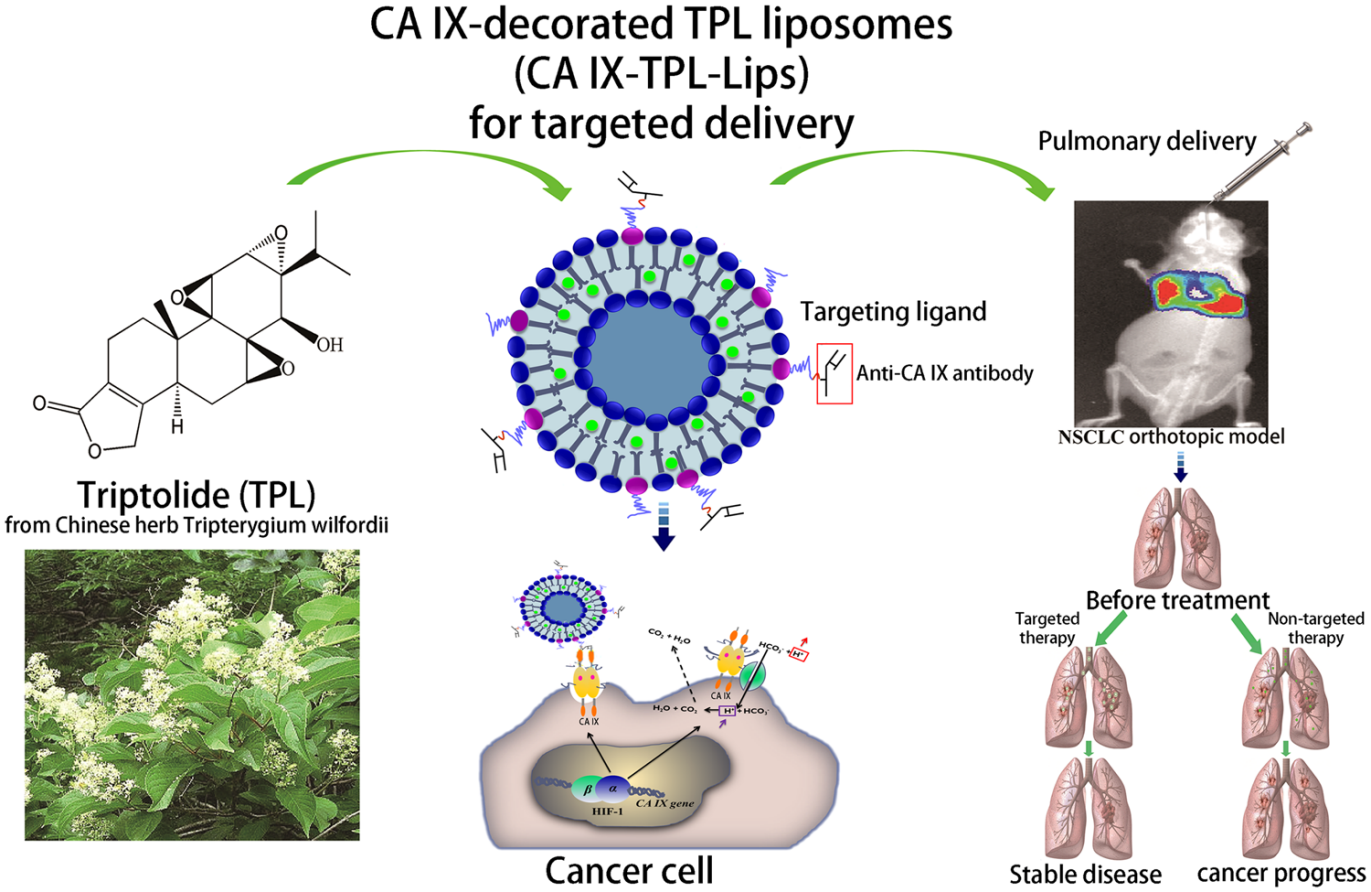
Schematic representation of CA IX-decorated TPL liposomes (CA IX-TPL-Lips) for lung cancer-targeted therapy by pulmonary delivery.

Based on the above rationale, we utilize the unique features of CA IX along with the anti-cancer properties of TPL and pulmonary delivery to develop a promising therapeutic formulation for NSCLC (Fig. 1). In this study, we modified liposomal surfaces with anti-CA IX antibody and encapsulated TPL for delivery via pulmonary route for lung cancer therapy. We analyzed various *in vitro* parameters of CA IX-TPL-Lips including particle size, drug encapsulation efficiency, drug release, stability, cellular uptake efficiency and cytotoxicity. The bio-distribution and therapeutic effect of CA IX-Lips were also examined in animal models carrying orthotopic lung tumors after endotracheal administration. This study provides insight into targeted and sustained delivery of a toxic drug through CA IX-Lips via the pulmonary route for lung cancer therapy.

## Results and Discussion

### Preparation and characterization of liposomal TPL

**Figure 2:**
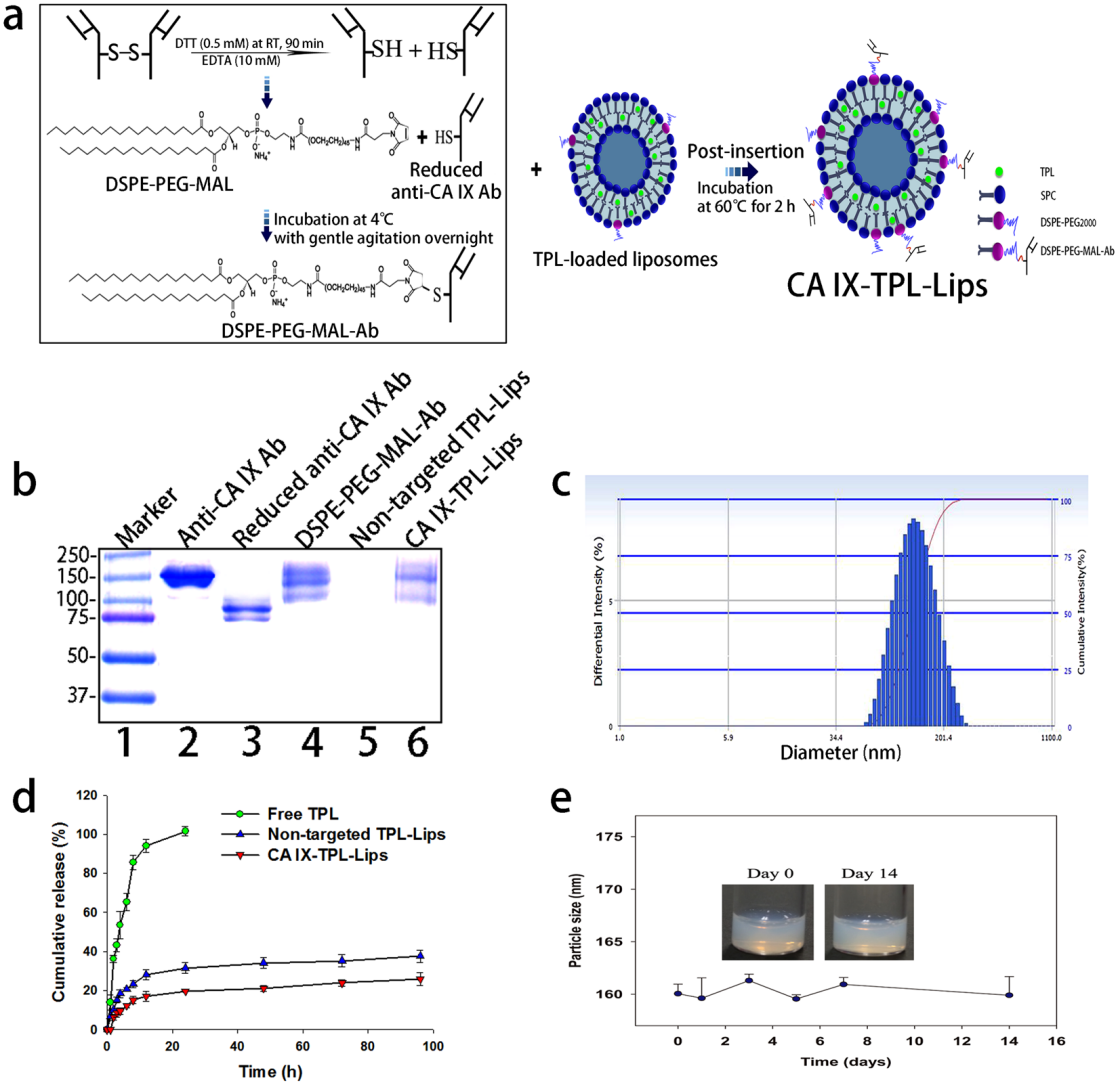
The preparation and characterization of CA IX-TPL-Lips. (**a**) Illustration of the preparation of CA IX-TPL-Lips; (**b**) Reducing SDS-PAGE electrophoresis of lane 1: molecular weight size marker, lane 2: Anti-CA IX antibody (Ab), lane 3: Reduced anti-CA IX antibody (Ab), lane 4: DSPE-PEG-MAL-Ab, lane 5: Non-targeted TPL-Lips and lane 6: CA IX-TPL-Lips; SDS-PAGE gel was stained with Coomassie Brilliant Blue R250 to visualize the Ab; (**c**) Representative particle size distribution of CA IX-TPL-Lips; (**d**) *In vitro* release profile of TPL formulations in PBS (pH 7.4); (**e**) Stability of CA IX-TPL-Lips at 4 °C evaluated by measuring the change in particle size.

Firstly, the antibodies were treated with the reducing agent dithiothreitol (DTT) at a mild condition to generate half-antibodies containing a free thiol group^30–32^ adequate for the formation of thioether with DSPE-PEG-maleimide (DSPE-PEG-MAL). Subsequently, we prepared CA IX-TPL-Lips by incorporating antibody-conjugated micelles into TPL-Lips (Fig. 2a). Incorporated liposomes were separated by Sepharose CL-4B gel filtration chromatography. The antibody we used in this study is a kind of immunoglobulin G (IgG), which contains two heavy chains and two light chains with intact molecular weight about 150 kDa^30^. After the reduction, half-antibody with a molecular mass around 75 kDa was generated, which contained an intact antigen binding site (heavy-light chain). The generated half-antibodies were verified with ultra-high performance liquid chromatograph with accurate mass quadrupole time-of-flight mass spectrometer (UPLC Q-TOF MS) (Fig. S1) and SDS-PAGE electrophoresis followed by Coomassie staining (Fig. 2b). The conjugation of reduced anti-CA IX antibody with DSPE-PEG-MAL micelles (DSPE-PEG-MAL-CA IX) and the successful preparation of CA IX-Lips were also confirmed by SDS-PAGE electrophoresis, demonstrated by the upper shift of the band due to the change in molecular weight (Fig. 2b). Protein smears observed in the lane of DSPE-PEG-MAL-CA IX and CA IX-TPL-Lips were probably due to the lipid content in the sample, which decreased the electrophoretic mobility of antibody chains^33^.

Particle size, polydispersity index, and entrapment efficiency of the prepared liposomal TPL are presented in Table 1. After incorporation of the antibody-conjugated micelles, the particle size increased significantly compared to non-targeted TPL-Lips, from 127.2 ± 4.93 nm to 160.1 ± 0.9 nm (p < 0.001), suggesting the presence of antibody molecules on the liposome surface. CA IX-TPL-Lips showed homogenous polydispersity index values and particle size distribution (Fig. 2c).

**Table 1:** Characterization of liposomal TPL.

Formulation	Particle size (nm)	Polydispersity index	EE (%)
Non-targeted TPL-Lips	127.2 ± 4.9	0.107 ± 0.047	85.6 ± 2.1
CA IX-TPL-Lips	160.1 ± 0.9^***^	0.175 ± 0.010	92.1 ± 1.8

Note: ***p < 0.001 when compared with the non-targeted TPL-Lips group.

The cumulative release profile of TPL from CA IX-Lips *in vitro* is shown in Fig. 2d. Both non-targeted TPL-Lips and CA IX-TPL-Lips revealed similar sustained release kinetics. The total cumulative release of non-targeted TPL-Lips and CA IX-TPL-Lips was approximately 37% and 25% within 96 h, respectively. These results revealed that the decoration with anti-CA IX antibody do not remarkably influence the release profile of the liposomes.

Furthermore, the CA IX-TPL-Lips were stable with no significant change in particle size and organoleptic features, such as aggregation and precipitation in 14 days (Fig. 2e).

### Cellular uptake and *in vitro* cytotoxicity of CA IX-Lips

Hypoxia is a salient feature of many types of solid tumors due to the increased metabolic production, impaired removal of CO_2_ and the abnormal vasculature microvasculature^34, 35^. Since CA IX is a hypoxia-inducible enzyme controlled by HIF, we cultured the cells under hypoxic conditions to mimic the *in vivo* microenvironment of solid tumors. Firstly, we confirmed that CA IX was expressed at high levels on the cell membrane of A549 cells after 24 h incubation under hypoxia (CA IX-positive), while no expression was seen under normoxia (CA IX-negative) (Supplementary Fig. S2). We applied hypoxic conditions to induce the expression of CA IX in A549 cells for the following *in vitro* experiment at cellular level.

**Figure 3:**
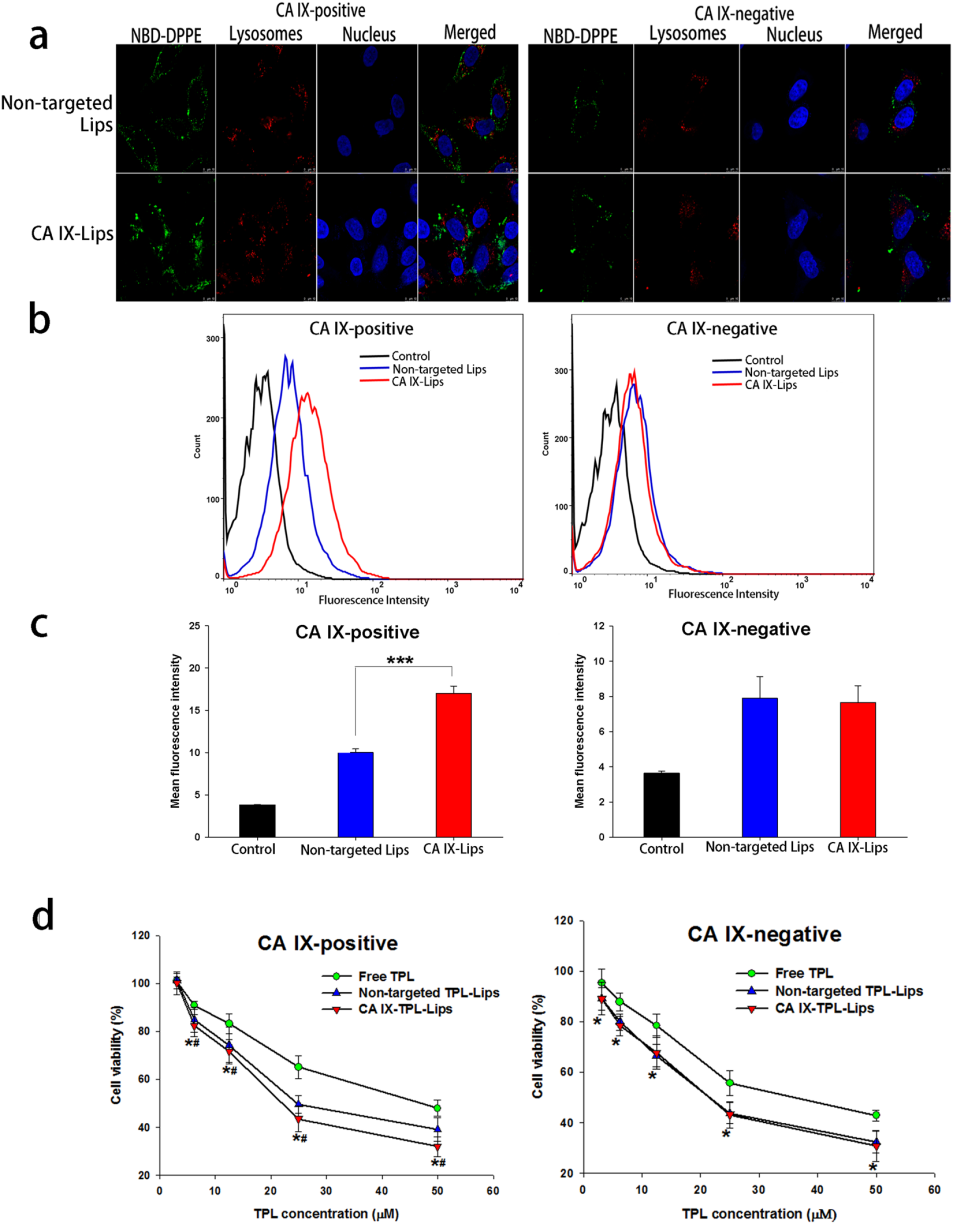
Cellular uptake and cytotoxicity of CA IX-TPL-Lips in NSCLC A549 cells. (**a**) Confocal laser scanning microscopy image of CA IX-negative and CA IX-positive A549 cells after treatment with NBD-DPPE labeled CA IX-Lips and non-targeted liposomes for 4 h; Scale bars, 10 μm. (**b**) Quantitative analysis of NBD-DPPE-labeled liposomes uptake by flow cytometry. (**c**) Mean fluorescence intensity of NBD-DPPE-labeled liposomes after 4 h treatment; control cells were untreated. Data are presented as mean ± SD (n = 3), ***Indicates p < 0.001. (**d**) *In vitro* cytotoxicity of free TPL, non-targeted TPL-Lips and CA IX-TPL-Lips against CA IX-positive and CA IX-negative A549 cells assessed by MTT assay. Data are presented as mean ± SD (n = 3). *p < 0.05 compared with free TPL group; ^#^p < 0.05 compared with non-targeted TPL-Lips group.

Next, we investigate the cellular uptake behavior of CA IX-Lips in CA IX-negative and positive A549 cells using confocal laser scanning microscope (CLSM; Leica TCS SP8, Leica Microsystems Ltd.). After 4 h incubation with NBD-DPPE-labeled liposomes, the CA IX modification enhanced the cellular uptake of liposomes in CA IX-positive cells (Fig. 3a). In contrast, in CA IX-negative cells, we found no difference between CA IX-decorated and non-targeted liposomes treated groups. The difference may be explained by the fact that modifying the liposomal surface with anti-CA IX antibody enhances the cell-binding affinity and accelerates internalization of the drug by immunobinding to CA IX protein.

We made quantitative measurements of the cellular uptake of NBD-DPPE-labeled liposomal formulations using flow cytometry. CA IX-positive A549 cells treated with CA IX-Lips showed a prominent right shift in the cytometric analysis compared to the non-targeted Lips, suggesting enhanced cellular uptake of the CA IX-Lips (Fig. 3b). The intracellular mean fluorescence intensity of CA IX-Lips was significantly increased in CA IX-positive cells (*p* < 0.001; Fig. 3c). However, there was no significant difference between CA IX-decorated and non-targeted liposomes in CA IX-negative cells. This result was consistent with the result from the qualitative study. Thus, CA IX-Lips had a greater targeting efficacy to CA IX-overexpressing cells than unmodified liposomes.

We then evaluated the *in vitro* efficiency of CA IX-TPL-Lips using an MTT assay. Following the induction of CA IX by hypoxia, the cell viability of CA IX-Lips treated group was significantly reduced compared to both non-targeted Lips group and free TPL group at TPL concentrations from 6.25 μM to 50 μM (Fig. 3d). For CA IX-positive cells, CA IX-Lips and non-targeted TPL-Lips both demonstrated a lower IC_50_ (14.67 μM and 29.09 μM) than the free TPL group (44.64 μM). Furthermore, the IC_50_ of CA IX-TPL-Lips was much lower than that of non-targeted TPL-Lips, which may be attributed to the improved targeting efficacy of the CA IX modification. However, we found no significant differences in cell viabilities of CA IX-negative cells when treated with the CA IX-decorated and non-targeted liposomal formulations at different concentrations, suggesting that the CA IX ligand played a key role in enhanced cytotoxicity *in vitro*.

### A549 tumor spheroids uptake

**Figure 4:**
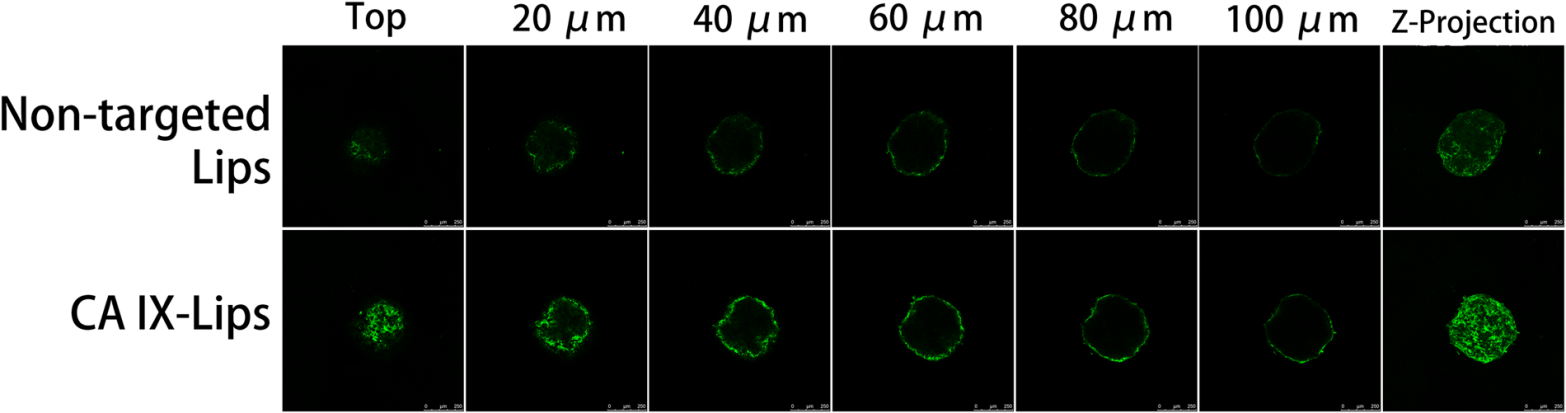
Uptake of NBD-DPPE-labeled CA IX-Lips and non-targeted Lips throughout A549 tumor spheroids. The distribution of liposomes were analyzed by CLSM using Z-stacking imaging with 20 μm intervals. Scale bars, 250 μm.

Compared to traditional monolayer culture system, the cells in multicellular spheroids are more closely similar those *in vivo* situation^36^. 3D culture conditions in the spheroids contain many of the characteristics of natural tissue including the production of an extracellular matrix, a core region of hypoxic and necrotic, which is demonstrated to provide a major barrier to traditional drugs and nanoparticles penetration in solid tumors. Thus, in the present work, A549 tumor spheroids were prepared to evaluate the solid tumor permeability of CA IX-Lips (Supplementary Fig. S3). Firstly, the expression of CA IX on tumor spheroids was confirmed by immunofluorescence (Supplementary Fig. S4). Then, in the uptake study, CA IX-Lips displayed stronger green fluorescence at different depth of A549 tumor spheroids compared to non-targeted Lips (Fig. 4). It is confirmed that CA IX-Lips possessed stronger penetration ability and binding affinity through anti-CA IX antibody modification.

### Establishment of orthotopic lung tumor models

**Figure 5:**
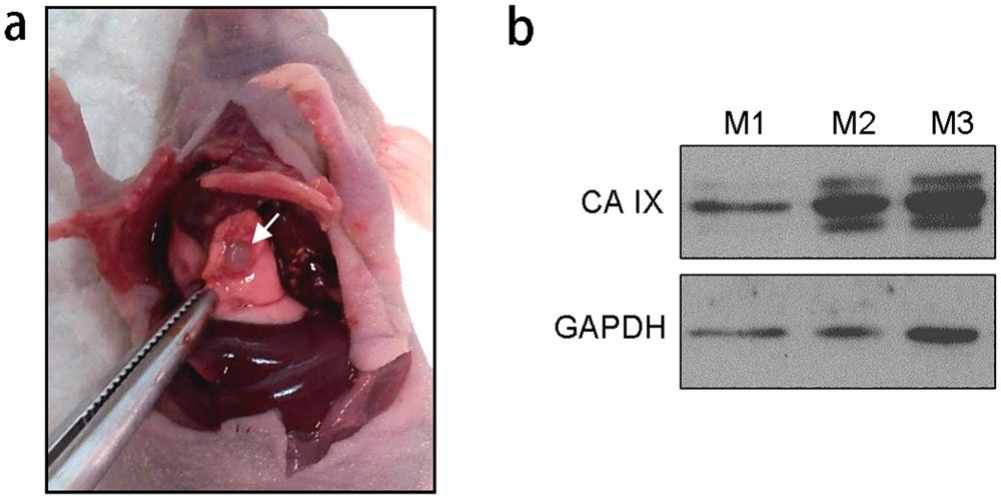
Establishment of orthotopic NSCLC tumor models. (**a**) Representative solitary tumor surrounded by normal lung tissue that was observed 56 days after intrathoracic injection of A549 cells; (**b**), Western blot detection of CA IX in A549 lung tumor xenografts collected from three individual mice (M1, M2, and M3). GAPDH was used as the loading control.

In this study, we demonstrated the therapeutic benefit of CA IX-decorated immunoliposomal TPL by using a mouse model of orthotopic NSCLC tumors. Many studies of lung cancer therapeutics have relied on ectopic tumor models generated by subcutaneous inoculation of human lung cancer cells into easily accessible body parts in mice. Organ microenvironment can in fact modulate the phenotype and sensitivity to chemotherapeutics of cancer cells^37, 38^. Orthotopic models in which tumors grow in the microenvironment of lung tissues thus provide more clinically relevant systems for the identification of novel anti-cancer drugs^39^. Here, the orthotopic mouse model of human NSCLC was developed by injecting A549 cells percutaneously into the thorax of the mouse. The implantation technique used in this study was simple and reproducible, with an operative mortality rate of only 4%. Matrigel was used as an anchor to fix the tumor cells at the site of injection and prevent unwanted cell spread. Figure 5a shows a representative solitary tumor mass observed within the lung 56 days after tumor cell implantation. CA IX expression due to hypoxic environment within the tumor was confirmed in excised tumor tissue samples (Fig. 5b). This mouse model can provide a clinically relevant system for studying the therapeutic efficacy of TPL delivered by CA IX-Lips.

### Bio-distribution of CA IX-Lips

**Figure 6:**
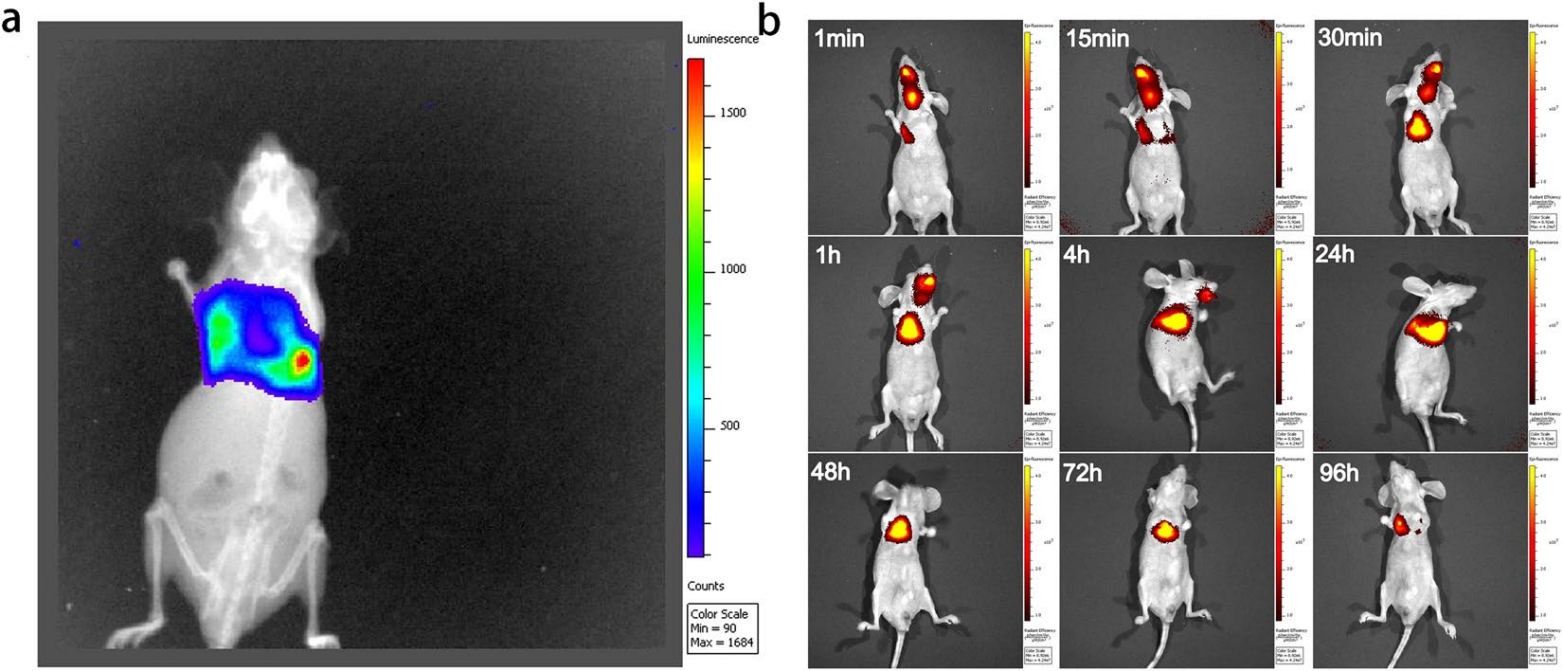
Bio-distribution behavior of CA IX-Lips. (**a**) Bioluminescence imaging of A549-Luc orthotopic tumor-bearing mouse; (**b**) Fluorescence imaging of CA IX-decorated DiR liposomes bio-distribution behavior in A549-Luc orthotopic tumor-bearing mouse at different time points after endotracheal administration.

Delivery route plays an important role in the treatment of NSCLC. Direct delivery of chemotherapeutics to the lung could provide a better accumulation of active ingredients in the lung and low systemic side effects^40^. In order to maximize exposure of lung tumor tissues to the drug and minimize the toxicity to normal organ tissue, the immunoliposomal drug was administrated via pulmonary delivery. The successful establishment of A549-Luc orthotopic lung cancer in the mouse was checked by bioluminescence imaging using the IVIS® Lumina XR system (Fig. 6a) before the bio-distribution study. Subsequently, the bio-distribution behavior of CA IX-Lips was investigated in the mouse by endotracheal administration. Figure 6b demonstrates that the CA IX-decorated DiR liposomes were only concentrated in the lung, and could even be seen at 96 h. This result could support the potential application of CA IX-decorated immunoliposomes as a sustained delivery system targeted at lung cancer site.

### *In vivo* anti-cancer efficacy

**Figure 7:**
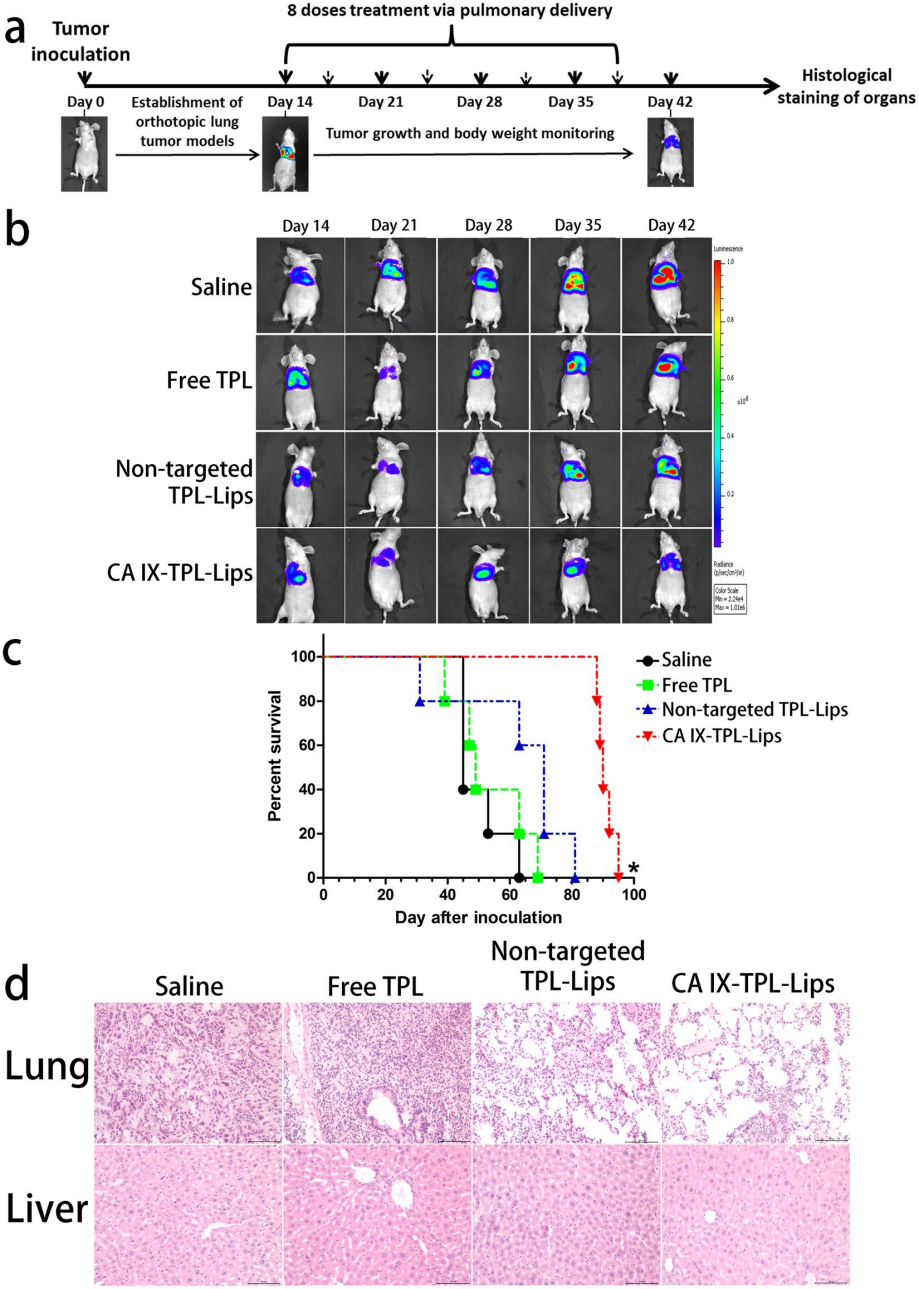
Anti-cancer effects of CA IX-TPL-Lips in mice with orthotopic lung tumors. (**a**) Treatment schedule of TPL formulations in orthotopic A549-Luc tumor-bearing BALB/c nude mice; (**b**) Representative serial images of bioluminescence change in mice during treatment with different formulations of TPL including CA IX-TPL-Lips, non-targeted TPL-Lips, free TPL solution and saline as a control (n = 5) using the IVIS® Lumina XR system. (**c**) Survival curve of mice with orthotopic lung tumors following administration of TPL formulations. *p < 0.05, compared with saline; (**d**) Histological staining of organs following pulmonary administration of TPL formulations. Scale bars, 100 μm.

Since TPL release from CA IX-Lips could be sustained for up to 96 h *in vitro*, and liposomes could be observed for up to 96 h in the bio-distribution study *in vivo*, the tumor-bearing mice were treated with TPL formulations twice a week. In the study, we monitored the variation in bioluminescence signals of the tumor and body weight to evaluate the anti-cancer efficacy of different TPL formulations in mice with orthotopic lung tumors (Fig. 7a). Figure 7b shows representative serial images of each group throughout the experimental period from day 14 to day 42 (4 days after the last dose of treatment). The group receiving CA IX-TPL-Lips exhibited the strongest tumor growth inhibition after 8 treatment doses compared to non-targeted TPL-Lips, free TPL and the saline groups. A change in body weight of tumor-bearing mice during treatment has often been used as a marker of toxicity. In the present study, no significant variations were observed in all treatment groups relative to the control group, indicating no obvious systemic toxicity of TPL formulations in pulmonary delivery (Supplementary Fig. S5). This result also demonstrates that pulmonary delivery of an anti-cancer drug can be a very well-tolerated strategy.

The lifespan of CA IX-TPL-Lips treated mice was significantly prolonged (*p* = 0.002 compared with control) (Fig. 7c) with a median survival time of up to 90 days, twice longer than in the control group. Although non-targeted TPL-Lips could prolong the median survival time from 45 days to 71 days, we found no significant difference between the saline group and non-targeted TPL-Lips group.

Representative histopathology samples are shown in Fig. 7d. In saline treated mice, tumors in the lung were composed of large cells with eosinophilic cytoplasm. We observed the typical features of tumor cells with large and irregular nuclei. The group receiving free TPL displayed highly condensed nuclei and peri-bronchial accumulation of chronic inflammatory cells. In the group treated with non-targeted liposomal TPL, small tumors appeared and were composed primarily of loose aggregates of large cells with foamy cytoplasm. Mice treated with CA IX-TPL-Lips presented lower density of tumor cells compared to the other groups. Tumors in the lungs of these animals were small and loosely organized and showed abundant foamy cytoplasm. In addition, because nanoparticles are generally taken up by the liver due to fenestrated endothelia and heavy blood flow in the systemic circulation^41, 42^, the use of TPL could result in serious adverse events mainly associated with the liver, such as hepatomegaly and hepatic injury^43^. We also studied histopathological changes of the liver in the present study. Only the free TPL caused slight damage to livers and no obvious hepatotoxicity was discovered in the liposome groups. This result demonstrates that pulmonary delivery decreases the toxicity of TPL to the liver.

The enhanced anti-cancer effect of the liposomal groups was mainly ascribed to the fact that the liposomes could offer prolonged time and sustained release of anticancer agents in the lung by pulmonary administration. By surface modification of liposomes with targeting ligands that bind specifically to these overexpressed receptors at tumor site and simultaneously administrated via pulmonary route, chemotherapeutic drugs can be delivered to the lung tumor sites and then released to the targeted cells. The anti-CA IX antibody on the liposomes could mediate the targeting of cancer cells by specific interaction with CA IX on the tumor cell surface, and thus improve tumor exposure to the cytotoxic drug. Results from the present study suggest the application of CA IX-TPL-Lips via pulmonary delivery for effective therapy of NSCLC.

## Conclusion

In the present study, a TPL-loaded, CA IX-decorated liposomes for drug delivery targeted at NSCLC with advantages in uniform particle size distribution and sustained released property was successfully developed. This work demonstrated that anti-CA IX antibody conjugated to liposomes could significantly increase cellular and tumor spheroids uptake to enhance the cytotoxicity of TPL in CA IX-positive cancer cells. Furthermore, *in vivo* studies showed that anti-CA IX antibody-decorated liposomal TPL not only suppressed tumor growth more efficiently than other non-targeted TPL formulations by pulmonary delivery, but also maximally extended the survival time of mice with orthotopic lung tumors. In summary, CA IX-Lips for TPL delivery via pulmonary route showed significant improvement in NSCLC therapy. Preclinical studies on a larger number of animal models for anti-cancer effect and comparison with intravenous administration are warranted. This work provides an insight for the development of active targeting liposome via pulmonary delivery to lung cancer treatment and the application of CA IX as a target antigen.

## Methods

### Animals

Male BALB/c nu/nu mice (7–8 weeks old) were obtained from the Laboratory Animal Services Center, the University of Hong Kong. All animal experiments were conducted according to the guidelines of the Committee on the use of Human and Animal Subjects in Teaching and Research of Hong Kong Baptist University and were approved by the Health Department of Hong Kong Special Administrative Region. Anesthesia was performed with an intraperitoneal injection of chloral hydrate at a dose of 300 mg/kg before experiments.

### Preparation of liposomal TPL

TPL-loaded liposomes (TPL-Lips) were prepared by the ethanol injection method^44^. Briefly, lipids containing SPC (360 mg) and DSPE-PEG_2000_ (40 mg) with or without TPL (4 mg) (at a lipid-to-drug weight ratio of 100:1) were dissolved in 0.5 mL of ethanol as organic phase and mixed thoroughly. The mixture was rapidly injected into 5 mL phosphate buffered saline (PBS, pH 7.4) under magnetic stirring at 60 °C for 1 h using a syringe needle. The obtained suspension was further passed once through a 0.2 μm pore size polycarbonate membrane and then five times through a 0.1 μm pore size membrane (Whatman, Maidstone, Kent, UK) under nitrogen gas using an extruder (Northern Lipids Inc., Burnaby, BC, Canada) to remove unincorporated drug aggregates and generate unilamellar vesicles of low polydispersity. NBD-DPPE/DiR-labeled liposomes were prepared as above, but with fluorescent dye instead of TPL.

### Incorporation of anti-CA IX antibody into preformed liposomes

Immunoliposomes were developed using the post-insertion technique with slight modification^44–46^. In brief, DSPE-PEG-MAL micelles were formed by hydrating the dried lipid film containing DSPE-PEG-MAL in PBS (pH 6.6, 0.01 M EDTA) at a concentration of 6 mM with heating at 65 °C for 30 min. The anti-CA IX antibody (MN, CA IX, 214274, USBiological life Sciences) was thiolated at the hinge region by reacting with the reducing agent^47^. The monoclonal anti-CA IX antibody was reduced with DTT (0.5 mM) at room temperature for 90 min in the presence of EDTA (10 mM) to obtain half antibody with a free sulfhydryl group^32^. The reducing agents from the reduced antibody mixture were immediately removed by ethyl acetate extraction. Subsequently, coupling of the antibody to the preformed micelles was carried out at a molar ratio of 1:60 by incubation at 4 °C with gentle agitation overnight. To prepare CA IX-TPL-Lips, antibody-conjugated micelles were incubated with preformed TPL-Lips (DSPE-PEG-MAL: SPC molar ratio of 1:82) at 60 °C for 2 h. In parallel, unmodified micelles were incubated with preformed TPL-Lips to obtain the non-targeted liposomes (non-targeted TPL-Lips). Sepharose CL-4B gel filtration was used to remove the unincorporated liposomes.

### The characterization of liposomes

The generation of half-antibodies by reducing agent was determined with UPLC Q-TOF MS and SDS-PAGE electrophoresis. The conjugation of the reduced anti-CA IX antibody to DSPE-PEG-MAL micelles (DSPE-PEG-MAL-CA IX) and the incorporation of the DSPE-PEG-MAL-CA IX into liposomes (CA IX-TPL-Lips) were checked by reducing SDS-PAGE electrophoresis followed by Coomassie staining.

After separation by Sepharose CL-4B, the particle size and distribution of the incorporated liposomes were determined by a Delsa Nano HC Particle Analyzer (Beckman Coulter, Brea, CA). For the measurement of entrapment efficiency (EE %), samples of the liposomal TPL preparations were taken and the unentrapped TPL was removed using the Amicon Ultra-0.5 centrifugal filter (10 K cutoff) by centrifugation (10000 rpm, 15 min). The concentration of the unentrapped TPL in the filtrate was quantified by ultra-performance liquid chromatography (UPLC, ACQUITY UPLC System, Waters, Milford, MA) using an ACQUITY UPLC BEH Shield RP18 column (1.7 μm, 2.1 mm × 100 mm, Waters) with UV detection at 230 nm. To determine the total amount of TPL in liposomes, 20 μL of the liposomes was ruptured with 980 μL of methanol. The injection volume was 2 μL, and the mobile phase consisted of water and acetonitrile containing 0.1% formic acid in a ratio of 70:30 (V:V) at a flow rate of 0.3 mL/min. EE (%) was calculated using the following Equation (1):

1 $${\rm{EE}}( \% )=\frac{Weight\,of\,entrapped\,triptolide}{Weight\,of\,total\,triptolide\,used}\times 100 \% $$

*In vitro* TPL release study was performed using the dialysis method. PBS (pH 7.4) was used as the release media. 1 mL of CA IX-TPL-Lips, non-targeted TPL-Lips or free TPL solution (0.05 mg/mL) were placed into dialysis tubes (MWCO 12–14 kDa) and tightly sealed. The dialysis tubes were immersed in 10 mL of PBS (pH 7.4) and incubated at 37 °C with gently shaking for 96 h. At predetermined time intervals, 100 μL release media was sampled and refilled with the same amount of fresh medium. The concentration of TPL was then determined by UPLC.

In addition, the stability of CA IX-TPL-Lips at 4 °C was monitored by measuring the change in particle size and organoleptic features, such as aggregation and precipitation.

### Expression of CA IX by A549 cells

A549 cells were cultured in Dulbecco’s modified Eagle’s medium (DMEM) with GlutaMAX supplemented with 10% fetal bovine serum, 100 U/mL penicillin, and 100 μg/mL streptomycin. The expression of CA IX on the surface of A549 cells was investigated under a normoxic or hypoxic condition^45^: A549 cells were exposed to normoxia (humidified air with 5% CO_2_) or hypoxia (in a Modular Incubator Chamber purged with 1% O_2_, 5% CO_2_ and balance N_2_) at 37 °C for 20 h, followed by CA IX detection using immunofluorescence analysis^48^. A549 cells were fixed with 4% cold paraformaldehyde for 15 min after incubation for 24 h under normoxic or hypoxic condition. Cells were washed three times with DPBS and blocked with 10% bovine serum albumin for 1 h at room temperature, then incubated with primary mouse monoclonal anti-CA IX antibody (20 μg/mL) overnight at 4 °C. Cells were washed three times followed by incubation with FITC-conjugated goat anti-mouse secondary antibody for 1 h at room temperature. A549 cells under normoxic or hypoxic condition, only treated with FITC-conjugated goat anti-mouse secondary antibody served as controls to avoid interference of cell auto-fluorescence. Nuclei were stained with Hoechst 33342 for 15 min after washing three times with DPBS. Cells were observed using a CLSM.

### Cellular uptake of CA IX-Lips

The cellular uptake of CA IX-Lips and non-targeted Lips after 4 h treatment was examined by CLSM. A549 cells were seeded at a density of 1 × 10^5^ onto a glass bottom dish, and grown overnight followed by incubation in normoxia or hypoxia for 24 h. Thereafter, the medium was replaced with serum-free medium and the cells were incubated with NBD-DPPE labeled CA IX-Lips or non-targeted Lips at a final NBD-DPPE concentration of 4 μg/mL for 4 h at 37 °C. 30 min before the treatment ended, LysoTracker Red was added to the cells at a final concentration of 50 nM. Cells were subsequently washed three times and fixed with 4% paraformaldehyde. The cells were washed three times with DPBS and treated with 2.5 μg/mL of Hoechst 33342 for 15 min at 37 °C to stain nuclei. Finally, the cells were washed three times with DPBS and visualized by CLSM.

For the quantitative study, A549 cells were seeded in 6-well plates at a density of 7.5 × 10^5^ cells per well and cultured for 24 h under normoxia or hypoxia. Different formulations of NBD-DPPE-labeled liposomes were added to the plates as described above. After 4 h incubation, the cells were washed three times with cold DPBS followed by trypsin treatment, and finally resuspended in 0.5 mL DPBS. The fluorescent intensity of the treated cells was determined using a FACSCanto flow cytometer (Becton Dickinson), acquiring 10,000 events per histogram.

### *In vitro* cytotoxicity assay

MTT assay was used to evaluate the anti-cancer effects of CA IX-TPL-Lips, non-targeted TPL-Lips and free TPL solution in CA IX-positive and CA IX-negative A549 cells. The cells were seeded in a 96-well multiwell plate at a density of 4 × 10^3^ cells per well and allowed to grow overnight. CA IX-positive and CA IX-negative cells were generated by parallel treatments with hypoxia and normoxia as described above. After 20 h, the medium was removed and the wells were washed twice with PBS. The cells were then incubated with various concentrations of TPL formulations and blank vesicles at 37 °C for 1 h. After removal of the unbound liposomes or free TPL, the cells were washed with PBS and then incubated with fresh medium at 37 °C for an additional 48 h. Next, 20 μL of MTT solution (5 mg/mL in PBS) was added to each well and incubated for another 4 h at 37 °C. Finally, MTT in medium was removed and 100 μL/well of DMSO was added to dissolve the formazan crystals. The absorbance was measured at 570 nm using a Benchmark Plus Microplate Reader (Bio-rad Laboratories).

### A549 tumor spheroids uptake

A549 tumor spheroids were firstly prepared as follows^49^: a 96-well plate was pre-coated with 2% (w/v) low melting point agarose in serum free DMEM medium; After cooling to solidify the agarose coating, 1 × 10^3^ A549 cells were seeded into each well and incubated for 7 days to form spheroids. CA IX expression of tumor spheroids was checked by immunofluorescence. For uptake study, the uniform and compacted spheroids were transferred to a confocal dish and incubated with NBD-DPPE labeled CA IX-Lips or non-targeted Lips for 4 h at 37 °C, then the spheroids were washed with cold DPBS and scanned began from the top to the equatorial plane to obtain the Z-stack images by CLSM.

### Establishment of orthotopic lung tumor models

Mice were implanted with A549 cells using the technique previously reported by Onn *et al*.^39^. A549 cell suspensions (1 × 10^7^ cells/mL) were prepared in Matrigel Matrix (4.35 mg/mL) (BD Biosciences, San Jose, CA). The mice (7–8 weeks) were anesthetized and placed in the right lateral decubitus position. 1 mL syringes with 29- gauge needles were used to inject 1 × 10^6^ cells percutaneously into the right lateral thorax, at the lateral dorsal axillary line, about 1.5 cm above the lower rib line just below the inferior border of the scapula. On day 56 after tumor cell implantation, three mice were sacrificed and lung tumor tissue was collected. Protein was extracted from tumor tissues using RIPA buffer with the Halt Protease and Phosphatase Inhibitor Cocktail. CA IX expression in tumor extract samples was confirmed by Western blot.

### Bio-distribution of CA IX-Lips

The bio-distribution and tumor-targeting properties of CA IX-Lips in A549-Luc orthotopic lung cancer BALB/c nude mice were investigated using a IVIS Lumina XR system (Caliper LifeSciences). A CA IX-decorated DiR liposomes suspension was administered directly via pulmonary delivery using a Microsprayer Aerosolizer Pulmonary Aerosol Kit for Mouse Model PAK-MSA (Penn-Century, Inc. Wyndmoor, PA19038 USA), and the distribution behavior in the nude mouse was monitored at different time points after endotracheal administration using an *in vivo* imaging system.

### *In vivo* anti-cancer efficacy

Orthotopic implantation of A549-Luc cells was performed in nude mice as described above. 14 days after tumor cell injection (day 14), mice were randomized into treatment and control groups (5 mice in each group). The treatment groups were given CA IX-TPL-Lips, non-targeted TPL-Lips and free TPL solution at a dose of 0.15 mg/kg^50^ once every 3–4 days for a total of 8 times via pulmonary delivery, while the control group received saline with the same conditions. Tumor growth was assessed by bioluminescence imaging once a week. D-luciferin substrate (Regis Technologies, Inc., Morton Grove, IL) was injected intraperitoneally at a dose of 150 mg/kg under anesthesia. Bioluminescence signals emitted from the implanted orthotopic A549-Luc tumors were measured 15 min later using the IVIS Lumina XR system. Body weights were monitored weekly throughout the study. The number of dead animals was recorded every day to create the survival curve. Lungs and livers were collected and applied for hematoxylin and eosin (H&E) staining.

### Statistical analysis

Data were expressed as mean ± standard deviation (SD), unless specified otherwise. Statistical analyses were performed using one-way repeated measures ANOVA with Student-Newman-Keuls test with Sigmaplot Software. The survival analysis was evaluated by the Kaplan-Meier method with GraphPad Prism 5 Software, followed by the log rank test for comparisons.

## Electronic supplementary material

Supplemental information

## References

[1] Siegel RL, Miller KD, Jemal A (2015). Cancer Statistics, 2015. Ca-Cancer J Clin.

[2] Garbuzenko OB (2010). Inhibition of lung tumor growth by complex pulmonary delivery of drugs with oligonucleotides as suppressors of cellular resistance. Proceedings of the National Academy of Sciences of the United States of America.

[3] Ramalingam SS, Owonikoko TK, Khuri FR (2011). Lung Cancer: New Biological Insights and Recent Therapeutic Advances. Ca-Cancer J Clin.

[4] Mura S, Nicolas J, Couvreur P (2013). Stimuli-responsive nanocarriers for drug delivery. Nature materials.

[5] Murakami, M. *et al*. Improving Drug Potency and Efficacy by Nanocarrier-Mediated Subcellular Targeting. *Sci Transl Med* **3**, doi:ARTN 64ra210.1126/scitranslmed.3001385 (2011). 10.1126/scitranslmed.3001385 21209412

[6] Allen TM (2002). Ligand-targeted therapeutics in anticancer therapy. Nat Rev Cancer.

[7] Chiche J (2009). Hypoxia-Inducible Carbonic Anhydrase IX and XII Promote Tumor Cell Growth by Counteracting Acidosis through the Regulation of the Intracellular pH. Cancer research.

[8] Vermylen P (1999). Carbonic anhydrase IX antigen differentiates between preneoplastic malignant lesions in non-small cell lung carcinoma. European Respiratory Journal.

[9] Swinson DEB (2003). Carbonic anhydrase IX expression, a novel surrogate marker of tumor hypoxia, is associated with a poor prognosis in non-small-cell lung cancer. J Clin Oncol.

[10] Le QT (2006). An evaluation of tumor oxygenation and gene expression in patients with early stage non-small cell lung cancers. Clinical Cancer Research.

[11] Liao SY, Aurelio ON, Jan K, Zavada J, Stanbridge EJ (1997). Identification of the MN/CA9 protein as a reliable diagnostic biomarker of clear cell carcinoma of the kidney. Cancer research.

[12] Saarnio J (1998). Immunohistochemical study of colorectal tumors for expression of a novel transmembrane carbonic anhydrase, MN/CA IX, with potential value as a marker of cell proliferation. Am J Pathol.

[13] Wykoff CC (2001). Expression of the hypoxia-inducible and tumor-associated carbonic anhydrases in ductal carcinoma *in situ* of the breast. Am J Pathol.

[14] Simi L (2006). Quantitative analysis of carbonic anhydrase IX mRNA in human non-small cell lung cancer. Lung Cancer.

[15] Pastorekova S (1997). Carbonic anhydrase IX, MN/CA IX: Analysis of stomach complementary DNA sequence and expression in human and rat alimentary tracts. Gastroenterology.

[16] De Simone G, Supuran CT (2010). Carbonic anhydrase IX: Biochemical and crystallographic characterization of a novel antitumor target. Bba-Proteins Proteom.

[17] Alterio V, Di Fiore A, D’Ambrosio K, Supuran CT, De Simone G (2012). Multiple binding modes of inhibitors to carbonic anhydrases: how to design specific drugs targeting 15 different isoforms?. Chem Rev.

[18] ClinicalTrials Database: NCT02215850. https://clinicaltrials.gov.

[19] Mahon BP, Pinard MA, McKenna R (2015). Targeting carbonic anhydrase IX activity and expression. Molecules.

[20] Siebels M (2011). A clinical phase I/II trial with the monoclonal antibody cG250 (RENCAREX(A (R))) and interferon-alpha-2a in metastatic renal cell carcinoma patients. World J Urol.

[21] Belldegrun, A. S. *et al*. ARISER: A randomized double blind phase III study to evaluate adjuvant cG250 treatment versus placebo in patients with high-risk ccRCC-Results and implications for adjuvant clinical trials. *J Clin Oncol* **31** (2013).

[22] Zatovicova M (2005). Ectodomain shedding of the hypoxia-induced carbonic anhydrase IX is a metalloprotease-dependent process regulated by TACE/ADAM17. Br J Cancer.

[23] Li, Y. Q., Shen, B. H., Kim, J. & Raz, D. Triptolide inhibits Wnt signaling due to DNA methylation alteration that is determined by dynamic histone 3 K79 lysine methylation in NSCLC. *Cancer research* **75**, doi:10.1158/1538-7445.AM2015-4777 (2015).

[24] Li XF (2016). Triptolide reduces proliferation and enhances apoptosis of human non-small cell lung cancer cells through PTEN by targeting miR-21. Mol Med Rep.

[25] Reno TA, Kim JY, Raz DJ (2015). Triptolide Inhibits Lung Cancer Cell Migration, Invasion, and Metastasis. Annals Of Thoracic Surgery.

[26] Zhou, Z. L., Yang, Y. X., Ding, J., Li, Y. C. & Miao, Z. H. Triptolide: structural modifications, structure-activity relationships, bioactivities, clinical development and mechanisms. *Nat Prod Rep* **29**, 457–475, doi:10.1039/c2np00088a (2012). 10.1039/c2np00088a 22270059

[27] Frese, S. *et al*. PG490-mediated sensitization of lung cancer cells to Apo2L/TRAIL-induced apoptosis requires activation of ERK2. *Oncogene* **22**, 5427–5435, doi:10.1038/sj.onc.1206842 (2003). 10.1038/sj.onc.1206842 12934102

[28] Lee KY, Park JS, Jee YK, Rosen GD (2002). Triptolide sensitizes lung cancer cells to TNF-related apoptosis-inducing ligand (TRAIL)-induced apoptosis by inhibition of NF-kappa B activation. Experimental And Molecular Medicine.

[29] Patil S (2015). Phosphonooxymethyl Prodrug of Triptolide: Synthesis, Physicochemical Characterization, and Efficacy in Human Colon Adenocarcinoma and Ovarian Cancer Xenografts. Journal of medicinal chemistry.

[30] Manjappa AS (2011). Antibody derivatization and conjugation strategies: application in preparation of stealth immunoliposome to target chemotherapeutics to tumor. J Control Release.

[31] Hutterer KM (2013). Monoclonal antibody disulfide reduction during manufacturing: Untangling process effects from product effects. mAbs.

[32] Mahmoud W (2011). Advanced procedures for labeling of antibodies with quantum dots. Analytical biochemistry.

[33] Moles E (2015). Immunoliposome-mediated drug delivery to Plasmodium-infected and non-infected red blood cells as a dual therapeutic/prophylactic antimalarial strategy. J Control Release.

[34] McIntyre A (2012). Carbonic Anhydrase IX Promotes Tumor Growth and Necrosis *In Vivo* and Inhibition Enhances Anti-VEGF Therapy. Clinical Cancer Research.

[35] McDonald PC, Winum JY, Supuran CT, Dedhar S (2012). Recent Developments in Targeting Carbonic Anhydrase IX for Cancer Therapeutics. Oncotarget.

[36] Mehta G, Hsiao AY, Ingram M, Luker GD, Takayama S (2012). Opportunities and challenges for use of tumor spheroids as models to test drug delivery and efficacy. J Control Release.

[37] Fidler IJ (1994). Modulation Of Tumor-Cell Response To Chemotherapy by the Organ Environment. Cancer Metast Rev.

[38] Wilmanns C, Fan D, Obrian CA, Bucana CD, Fidler IJ (1992). Orthotopic And Ectopic Organ Environments Differentially Influence the Sensitivity Of Murine Colon-Carcinoma Cells To Doxorubicin And 5-Fluorouracil. Int J Cancer.

[39] Onn A (2003). Development of an orthotopic model to study the biology and therapy of primary human lung cancer in nude mice. Clinical Cancer Research.

[40] Luo T (2016). PEGylation of paclitaxel largely improves its safety and anti-tumor efficacy following pulmonary delivery in a mouse model of lung carcinoma. J Control Release.

[41] Owens DE, Peppas NA (2006). Opsonization, biodistribution, and pharmacokinetics of polymeric nanoparticles. Int J Pharm.

[42] Chaudhari KR (2012). Opsonization, Biodistribution, Cellular Uptake and Apoptosis Study of PEGylated PBCA Nanoparticle as Potential Drug Delivery Carrier. Pharmaceutical research.

[43] Zhang ZR (2009). The targeting of 14-succinate triptolide-lysozyme conjugate to proximal renal tubular epithelial cells. Biomaterials.

[44] Pons M, Foradada M, Estelrich J (1993). Liposomes Obtained by the Ethanol Injection Method. Int J Pharm.

[45] Wong BCK (2014). Carbonic anhydrase IX-directed immunoliposomes for targeted drug delivery to human lung cancer cells *in vitro*. Drug Des Dev Ther.

[46] Iden DL, Allen TM (2001). *In vitro* and *in vivo* comparison of immunoliposomes made by conventional coupling techniques with those made by a new post-insertion approach. Bba-Biomembranes.

[47] Urban P, Estelrich J, Cortes A, Fernandez-Busquets X (2011). A nanovector with complete discrimination for targeted delivery to Plasmodium falciparum-infected versus non-infected red blood cells *in vitro*. J Control Release.

[48] Wei T (2013). Functionalized Nanoscale Micelles Improve Drug Delivery for Cancer Therapy *in Vitro* and *in Vivo*. Nano Lett.

[49] Liu YY (2014). Paclitaxel loaded liposomes decorated with a multifunctional tandem peptide for glioma targeting. Biomaterials.

[50] Fidler JM, An JH, Carter BZ, Andreeff M (2014). Preclinical antileukemic activity, toxicology, toxicokinetics and formulation development of triptolide derivative MRx102. Cancer Chemoth Pharm.

