# Supplementary material for: Pulmonary delivery of triptolide-loaded liposomes decorated with anti-carbonic anhydrase IX antibody for lung cancer therapy
Source: Sci Rep. 2017 Apr 20;7:1097. doi: 10.1038/s41598-017-00957-4 (PMC5430522; doi:10.1038/s41598-017-00957-4)
Supplement: Supplementary file 1 — Supplemental information [file 41598_2017_957_MOESM1_ESM.pdf]

Supplemental information

Pulmonary delivery of triptolide-loaded liposomes decorated with anti-carbonic anhydrase IX antibody for lung cancer therapy

Congcong Lin[1], Blenda Chi Kwan Wong[1], Hubiao Chen[1], Zhaoxiang Bian[1], Ge Zhang[1], Xue Zhang[1], Muhammad Kashif Riaz[1], Deependra Tyagi[1], Ge Lin[3], Yanbo Zhang[4], Jinjin Wang[2], Aiping Lu[1,2,*], Zhijun Yang[1,2,*]

[1]School of Chinese Medicine, Hong Kong Baptist University, 7 Baptist University Road, Kowloon Tong, Hong Kong, China

[2] Changshu Research Institute, Hong Kong Baptist University, Changshu Economic and Technological Development (CETD) Zone, Changshu 215500, China

[3] School of Biomedical Sciences, Chinese University of Hong Kong, Area 39, CUHK, Shatin, NT, Hong Kong, China

[4] School of Chinese Medicine, Li Ka Shing Faculty of Medicine, The University of Hong Kong, 10 Sassoon Road, Pokfulam, Hong Kong, China

* Corresponding author.

Correspondence and requests for materials should be addressed to Z.Y. (email: yzhijun@hkbu.edu.hk; Tel.: +852-3411-2961; fax: +852-34112461)

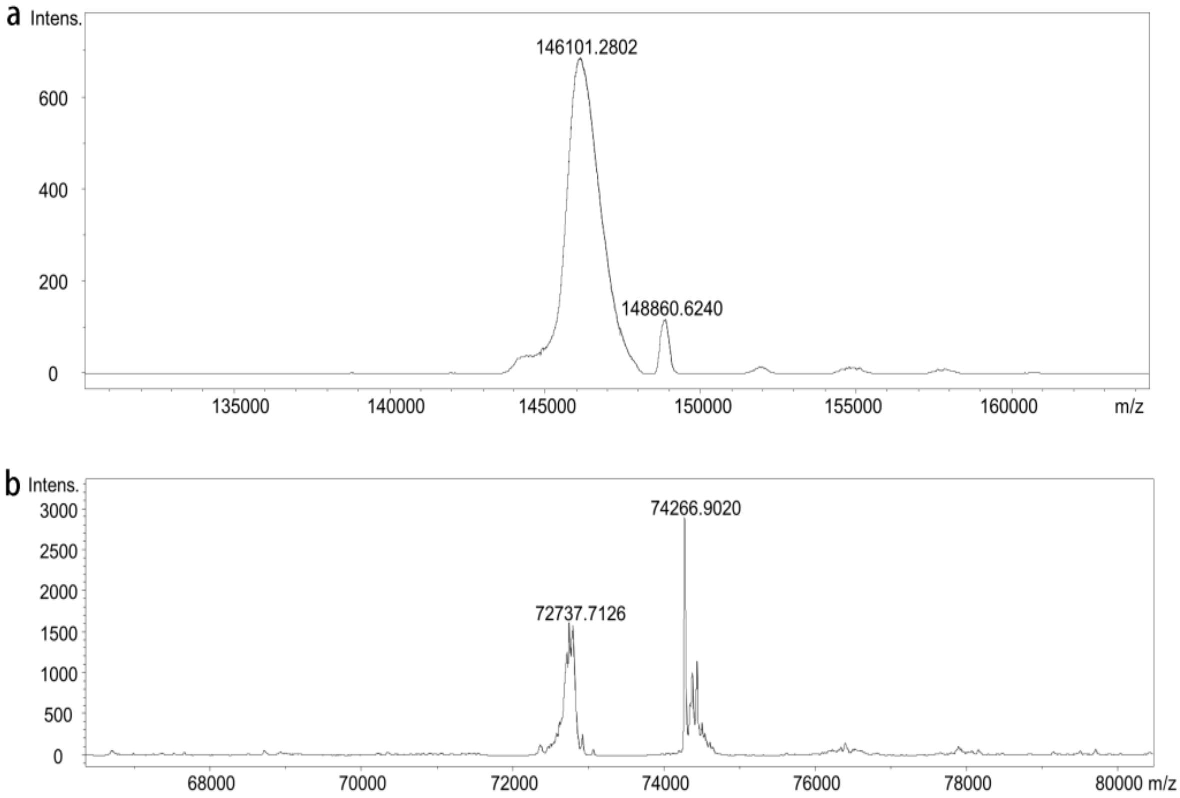

Figure S1. UPLC Q-TOF MS spectra of (a) Anti-CA IX antibody and (b) Reduced anti-CA IX antibody.

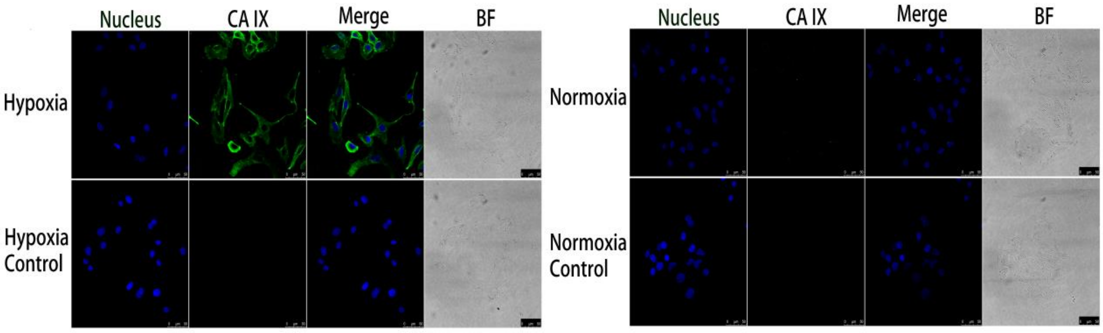

Figure S2. Expression of CA IX on cell membranes detected with FITC-conjugated goat anti-mouse secondary antibody (green) by confocal laser scanning microscopy. The control cells were not pretreated with primary monoclonal anti-CA IX antibody, but only treated with FITC-conjugated secondary antibody. Nuclei were stained with Hoechst 33342 (blue).

Scale bars, 50 μm.

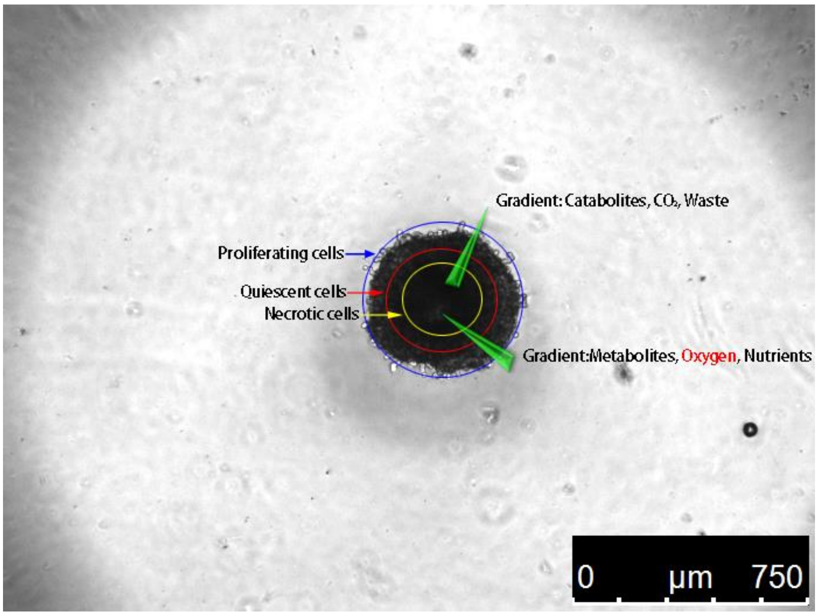

Figure S3. Brightfield image of A549 tumor spheroid captured by optical microscope with pathophysiological gradients schematically reported.

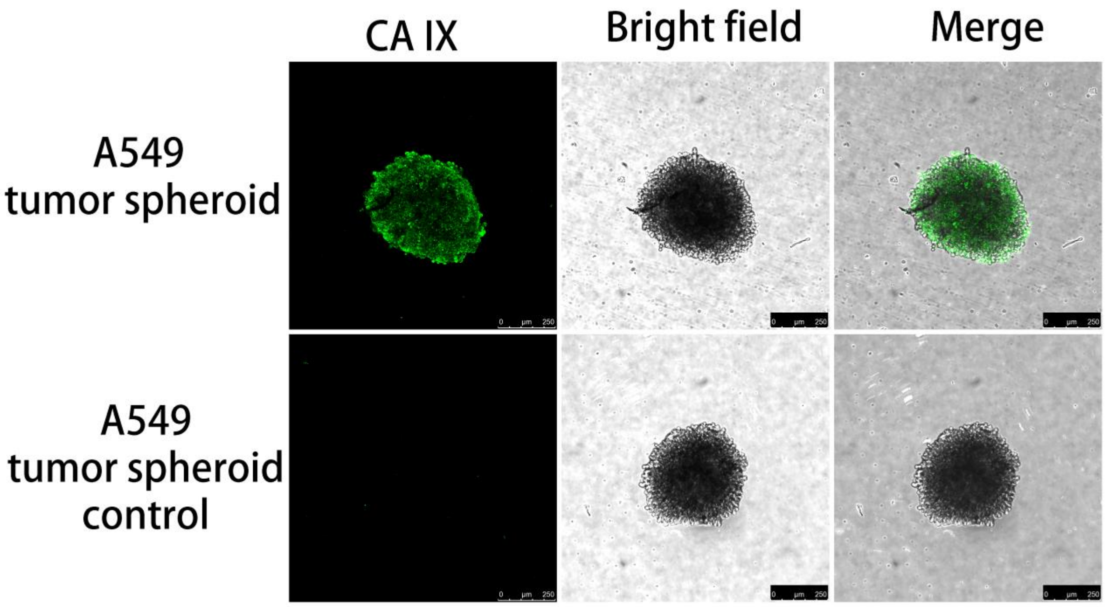

Figure S4. Expression of CA IX in A549 3D tumors. Confocal images of CA IX expression, detected with FITC-conjugated goat anti-mouse secondary antibody (green); The control A549 tumor spheroid were not pretreated with primary monoclonal anti-CA IX antibody, but only treated with FITC-conjugated secondary antibody. Scale bars, 250 μm.

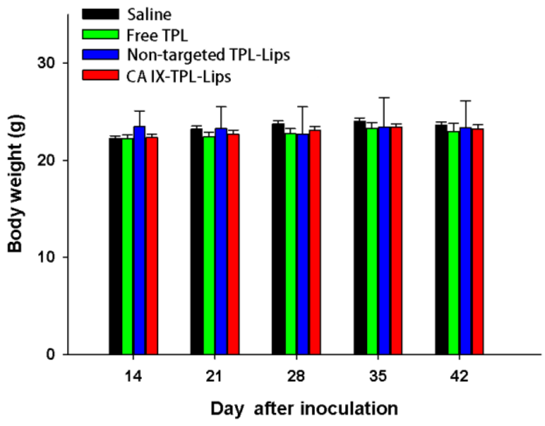

Figure S5. Body weights of mice receiving TPL formulations intratracheally.
